# Potential of Colostrum-Derived Exosomes for Promoting Hair Regeneration Through the Transition From Telogen to Anagen Phase

**DOI:** 10.3389/fcell.2022.815205

**Published:** 2022-03-10

**Authors:** Hyosuk Kim, Yeongji Jang, Eun Hye Kim, Hochung Jang, Haeun Cho, Geonhee Han, Hyun Kyu Song, Sun Hwa Kim, Yoosoo Yang

**Affiliations:** ^1^ Center for Theragnosis, Biomedical Research Division, Korea Institute of Science and Technology, Seoul, South Korea; ^2^ Department of Life Science, Korea University, Seoul, South Korea; ^3^ Division of Bio‐Medical Science and Technology, KIST School, Korea University of Science and Technology, Seoul, South Korea; ^4^ Department of Biotechnology, Korea University, Seoul, South Korea; ^5^ KU-KIST Graduate School of Converging Science and Technology, Korea University, Seoul, South Korea

**Keywords:** colostrum, exosome, hair growth, lactoferrin, dermal papilla

## Abstract

Human hair dermal papillary (DP) cells comprising mesenchymal stem cells in hair follicles contribute critically to hair growth and cycle regulation. The transition of hair follicles from telogen to anagen phase is the key to regulating hair growth, which relies heavily on the activation of DP cells. In this paper, we suggested exosomes derived from bovine colostrum (milk exosomes, Milk-exo) as a new effective non-surgical therapy for hair loss. Results showed that Milk-exo promoted the proliferation of hair DP cells and rescued dihydrotestosterone (DHT, androgen hormones)-induced arrest of follicle development. Milk-exo also induced dorsal hair re-growth in mice at the level comparable to minoxidil treatment, without associated adverse effects such as skin rashes. Our data demonstrated that Milk-exo accelerated the hair cycle transition from telogen to anagen phase by activating the Wnt/β-catenin pathway. Interestingly, Milk-exo has been found to stably retain its original properties and efficacy for hair regeneration after freeze-drying and resuspension, which is considered critical to use it as a raw material applied in different types of alopecia medicines and treatments. Overall, this study highlights a great potential of an exosome from colostrum as a therapeutic modality for hair loss.

## Introduction

Hair loss, a type of non-scarring alopecia, is characterized by defects in and loss of hair progenitor cells, and it occurs when hair follicles become smaller due to the influence of androgenic hormone (e.g., dihydrotestosterone, DHT). Although about 80% of Caucasian men is known to experience hair loss by the age of 70, only two androgenic alopecia treatments (minoxidil; potassium channel opener, and finasteride; inhibitor of type II 5α-reductase) are approved by the US Food and Drug Administration (FDA) (Rathnayake and Sinclair, 2010; Kelly et al., 2016). In addition, their effects are often limited and temporary, and they are also associated with various adverse effects (Wu et al., 2016; Locci and Pinna, 2017).

**TABLE 1:** Criteria for quantification of hair regeneration area.

No change
Under 30% shaved area with skin darkening and no hair growth
30–70% of shaved area with skin darkening and no hair growth
Above 70% of shaved area with skin darkening or under 30% showing hair growth
Above 70% shaved area with skin darkening and 30–70% showing hair growth
Above 70% shaved area with skin darkening, and above 70% showing hair growth
Above 90% shaved area with hair growth

All hair follicles periodically undergo a hair cycle that consists of anagen (growth), catagen (regression) and telogen (rest) (Ji et al., 2021). Many recent studies have reported that hair cycle is regulated by the interaction between mesenchymal cells and epithelial cells in hair follicles, and the proliferation of dermal papilla (DP) cells by the activation of PI3K/Akt/Wnt/β-catenin pathway may be the key factor (Botchkarev and Kishimoto, 2003; Kang et al., 2020).

Based on the knowledge of the hair follicle cycle, recent treatment modalities for hair loss attempt to convert hair follicles from a resting phase (telogen) to a growth phase (anagen) by activating hair follicle stem cells or attempt to make dermal papilla cells from the hair follicle stem cells (Ji et al., 2021). Providing adequate signals and environment to reactivate hair follicle stem cells and regrow a hair follicle is of particular interest to hair regeneration. In this regard, here we investigated the potential of exosomes derived from colostrum as a future hair regrowth therapy.

Exosomes are tiny extracellular vesicles (EVs, ∼ 50–200 nm) that play an important role in communicating with each other between cells in the body. Exosomes contain cargoes of proteins (e.g., cytokines, growth factors), lipids, nucleic acids such as miRNA (microRNA), which deliver chemical information to other cells. In particular, exosomes have been demonstrated as important modulators of paracrine signaling. For example, DP cell-derived exosomes or EVs derived from mesenchymal stem cells have been reported to be of major importance for hair follicle regeneration (Rajendran et al., 2017; Kwack et al., 2019; Hu et al., 2020). Despite favorable outcomes from many of the preclinical studies, however, no clinical studies were reported currently employing extracellular vesicle or exosome therapy for hair growth. The limited clinical application seems to be due to the high production cost and requirement of specialized purification methods associated with their short shelf life. In addition, the administration of stem cell-derived exosomes carries the potential risks of the uncontrolled transmission of genetic information and immune responses (Ma et al., 2020).

Exosomes can be produced and collected from cultured cells, but they also can be found in nearly all body fluids including breast milk. In particular, bovine milk can generate massive amounts of exosomes cost-effectively compared to exosomes derived from cell lines or other biological fluids (Somiya et al., 2018; Zempleni et al., 2019). These exosomes are highly appreciated for therapeutic applications in terms of safety. In addition, milk exosomes, especially from colostrum, have been found to contain over 1,000 growth factors and biological signals like FGF, PDGF, VEGF, and many others that were critical for promoting and accelerating healing, tissue regeneration (Samuel et al., 2017; Yang et al., 2017). Beyond the importance of bovine colostrum for the calf’s ability to resist infections and diseases, the value of bovine colostrum as a therapeutic modality has now been documented in several clinical trials (Struff and Sprotte, 2008).

**FIGURE 1:**
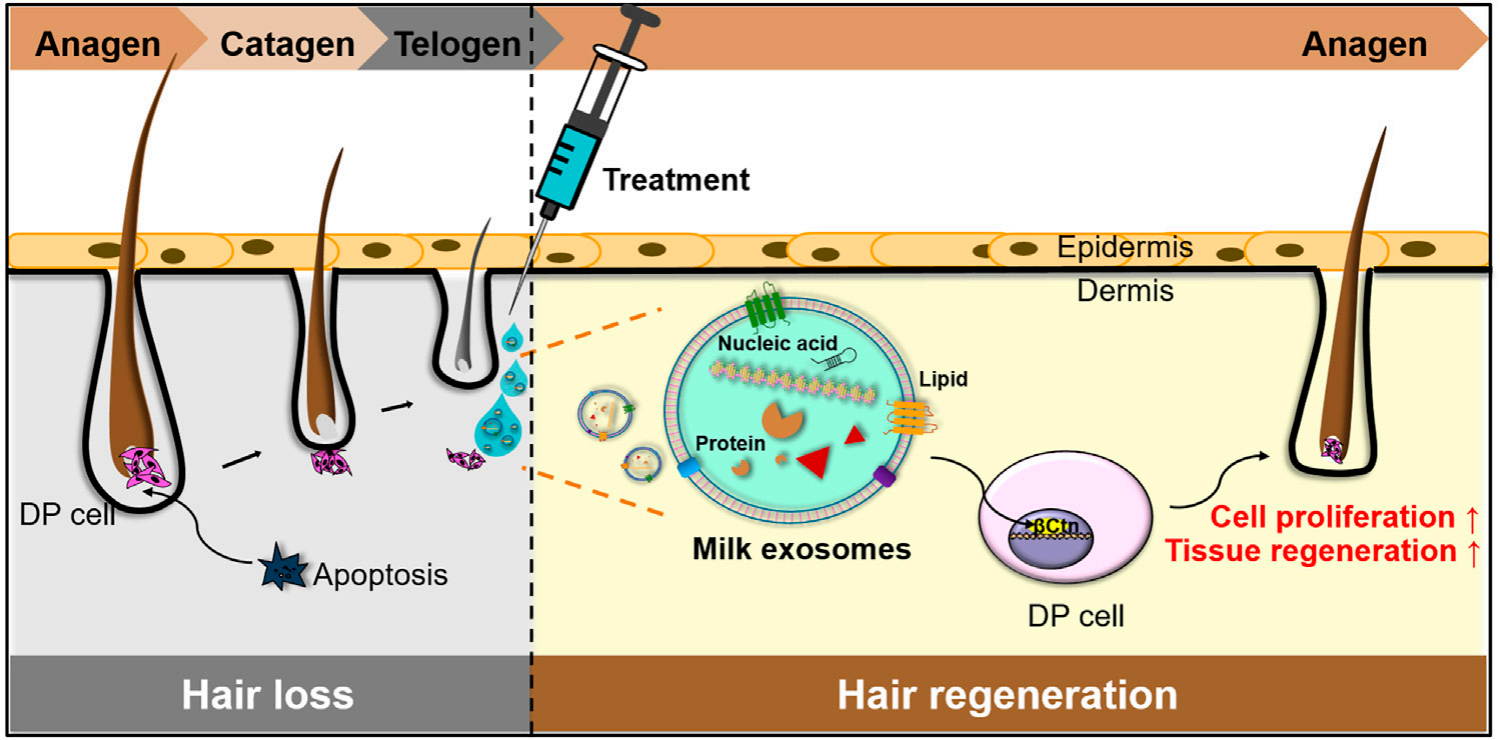
Schematic illustration of Milk-exo-mediated phase transition from telogen to anagen for hair regeneration.

Here we provide evidence that milk exosomes from colostrum (Milk-exo), induce impressive hair regeneration with minimal adverse effects (Figure 1) We found that Milk-exo induced the proliferation of DP cells that plays a pivotal role in controlling the growth and cycling of hair follicles. Moreover, Milk-exo overcame DHT-induced cell cycle arrest of DP cells and improved mouse dorsal hair growth with increased expression of *β*-catenin in hair follicles. Results showed that Milk-exo accelerated the conversion of telogen to anagen phase by activating the Wnt signaling system, which is involved in the hair growth cycle and the activation of the hair follicle stem cells (Huelsken et al., 2001; van Amerongen and Nusse, 2009). Collectively, we believe that milk exosomes from colostrum could provide a less-invasive cell-free treatment option for patients with hair loss.

## Material and Methods

### Isolation and Characterization of Milk Exosomes From Colostrum

Colostrum from healthy cows was purchased from a ranch named Ha-yan after routine milking. Milk exosomes from colostrum (Milk-exo) and exosome free fraction of milk (Exo-free milk) were isolated by ultracentrifugation and filtration. All centrifugations were performed at 4°C. Milk was centrifuged at 5,000 g for 30 min, 12,000 g for 1 h to remove milk fat globules, somatic cells, and cell debris. This defatted milk was stored at -80°C until use. After the milk was thawed, milk was then subjected to serial centrifugation steps at 35,000 g for 1 h, 70,000 g for 3 h using an Optima XE-100 Ultracentrifuge (Beckman Instruments Inc., Palo Alto, CA, USA) to remove residual milk fat globules, casein. The supernatant was filtered through 0.80, 0.45, and 0.2 μm filters (Sartorius, Gottingen, Germany). The filtered supernatant was ultracentrifuged at 100,000 g for 1 h. The obtained exosomal pellet was resuspended in cold PBS. The supernatant was further depleted from exosomes by ultracentrifugation at 200,000 g for 3 h. After the last ultracentrifugation, the remaining Exo-free milk supernatant was used for comparison purposes. Pierce™ BCA protein assay kit (Thermo Fisher, Waltham, MA, USA) was used for quantifying the total protein concentration of Milk-exo and Exo-free milk. The size distribution of Milk-exo and Exo-free milk was obtained by dynamic light scattering (DLS) using Zetasizer Nano ZS (Malvern Instruments, United Kingdom). For DLS measurements, Milk-exo and Exo-free milk (100 μg) were diluted in 1 ml of PBS and then transferred to a disposable cuvette. The mean values of particle sizes were obtained from more than three independent preparations. The structures of Milk-exo were detected by transmission electron microscope (TEM) (Tecnai F20 G2). 10 μg of Milk-exo dissolved in PBS was transferred to an electron microscope grid and 1% uranyl acetate solution was used for negative staining.

### Western Blot Analysis

Milk-exo and Exo-free milk were quantitated by bicinchoninic acid (BCA) protein assays, and the proteins were separated with 8–12% sodium dodecyl sulfate polyacrylamide gel and transferred to nitrocellulose membrane using a Trans-Blot Turbo (Bio-Rad, Hercules, CA, USA). The membrane was blocked in 5% skim milk for 1 h at room temperature followed by incubating with primary antibodies at 4°C overnight: anti-Tsg101 (1:1000, Abcam, Cambridge, MA, USA), anti-Alix (1:500, Novus Biologicals, Littleton, CO, USA), anti-MFG-E8 (1:1000, R&D Systems, Wiesbaden-Nordenstedt, Germany), anti-GM130 (1:1000, Invitrogen™ Thermo Fisher Scientific, Waltham, MA, USA), anti-Lactoferrin (1:1000, Santa Cruz Biotechnology, Dallas, TX, USA), anti-Wnt3a (1:1000, Abcam), anti-β-catenin (1:500, Abcam), GAPDH (1:1000, R&D Systems). After 3 times wash with TBST (Tris-buffered saline, 0.1% Tween 20), the membranes were incubated with horseradish peroxidase (HRP)-conjugated secondary antibodies for 1 h at room temperature. The protein bands were visualized with iBright™ CL750 Imaging System (Invitrogen™ Thermo Fisher Scientific, Waltham, MA, USA).

### Cell Culture

Human follicle dermal papilla cells (PromoCell, Heidelberg, Germany) were cultured in follicle dermal papilla cell medium (PromoCell, Heidelberg, Germany) with fetal calf serum (0.04 ml/ml), bovine pituitary extract (0.004 ml/ml), basic fibroblast growth factor (1 ng/ml), insulin (5 μg/ml), and 1% antibiotic antimycotic (Gibco, Darmstadt, Germany). The passage 4–6 cells were maintained in 5% CO₂ at 37°C and subcultured when the cells reached 70–80% confluency. The medium was replaced with fresh medium every 3 days.

### Cellular Uptake of Milk-Exo

DP cells (5 × 10^4^) were seeded on a 35 mm glass-bottom confocal dish (SPL Life Science). After 12 h incubation, the medium was removed and washed with DPBS (Welgene, Seoul, Korea). For the fluorescent labeling of exosomes, Milk-exo was incubated in a 65 μM Cyanine 5.5-NHS ester (BioActs, Incheon, Korea) staining solution for overnight at 4°C, and free dye was removed using air-fuge (Beckman Instruments Inc., Palo Alto, CA, USA). After centrifugation for 1 h, washed with PBS and centrifugation again. The labeled exosomal pellet was resuspended in PBS. The labeled exosomes (100 μg) were then added into the dish and incubated in 5% CO₂ at 37°C for 1, 12, and 24 h. After incubation, cells were washed with DPBS and fixed in 4% paraformaldehyde for 10 min. The nucleus of cells was stained with 4’, 6-Diamino-2-Phenylindole (DAPI) (Sigma Aldrich, St. Louis, MO, USA). Images were analyzed with a confocal microscope Leica TCS SP6 (Leica Microsystems, Wetzlar, Germany).

### Proliferation Assay of DP Cells

The Cell-Counting Kit 8 (Dojindo Laboratories, Kumamoto, Japan) was used for cell proliferation assay. DP cells (7.5 × 10^3^/well) were seeded on a 96–well plate and cultured in the complete medium. After 12 h incubation, the medium was removed from each well. Cells were washed in DPBS and treated with various concentrations of Milk-exo (50, 100, 200, 300, 400, 500, and 600 μg/ml) or Exo-free milk (400 μg/ml) for 24 h in serum-free medium. CCK-8 solution was added, and absorbance at 450 nm was measured with a SpectraMax 34 microplate reader (Molecular Devices, Sunnyvale, CA, USA). To measured DP cell proliferation against DHT, DP cells (7.5 × 10^3^) were seeded on a 96-well plate and cultured in the complete medium. After 12 h incubation, the medium was removed from each well. Cells were washed in DPBS and treat with 30 μM DHT solution for 24 h in a serum-free medium. The cells were then added with Milk-exo or Exo-free milk (400 μg/ml). After 24 h, CCK-8 solution was added, and the optical density was measured at 450 nm.

### Animals and *In Vivo* Studies

All animal experiments were conducted in accordance with the International Guide for the Care and Use of Laboratory Animals and approved by the Korea Institute of Science and Technology. C57BL/6 mice (male, 7 weeks old) were purchased from Orient Bio Animal Center (Seongnam, Korea) and maintained standard habitat conditions for 1 week.

To determine the optimal time interval, the biodistribution study was performed using labeled Milk-exo as described above. Animals were intradermally injected into five spots (20 μL per site) with saline or 100 μg Milk-exo in 100 μL saline was intradermally injected into five spots (20 μL per site) on the dorsal skin of C57BL/6 mice (*n* = 3). The fluorescence intensity was analyzed using an IVIS Lumina Series Ⅲ *in vivo* imaging system (PerkinElmer, MA, USA). 24 h after injection, mice were sacrificed, and the skin and other major organs were collected. The IVIS Lumina series Ⅲ imaging system was used to measure fluorescence intensities of skin and organs. Quantification of fluorescence intensities was analyzed using Living Image software (PerkinElmer, MA, USA).

To determine whether Milk-exo could induce hair growth, we examined the effect of Milk-exo in C57BL/6 mice model. After acclimatizing, mice (*n* = 12) were randomly divided into four groups. The dorsal hair was shaved using animal clippers and applied with hair removal cream to observe the pink skin. Animals were intradermally injected into five spots (20 μL per site) with saline, 200 μg Milk-exo in 100 μL saline, and 200 μg Exo-free milk in 100 μL saline. 2.5% minoxidil was topically applied as a positive control. Each treatment was applied every other day for 19 days. Hair regeneration was assessed for each animal following the assignment of a hair regeneration scoring, which also assessed skin color and hair area. Mice were imaged following treatment and quantification of hair regeneration area was assessed using ImageJ based on the criteria below.

### Histological Examination

After sacrificing the mice to collect the dorsal skin, the skin tissue was fixed with 4% paraformaldehyde and washed with PBS. Fixed skin samples were dehydrated through serial graded ethanol, cleared in xylene, and embedded in paraffin to obtain longitudinal sections. The paraffin blocks were cut longitudinally into 7 µm thick sections. To observe the histological change, sections were stained with hematoxylin and eosin (H&E). Briefly, sections were deparaffinized in xylene for 30 min, hydrated in serial graded ethanol (100%, 90%, 80% and 70%) and then stained with hematoxylin for 1 min, followed by washes for 15 min and eosin staining for 15 s. They also dehydrated three times for 2 min in graded ethanol (70, 80, 90 and 100%) stepwise. They mounted the cover glass after cleaning with xylene. The number of hair follicles was counted in the deep subcutis (*n* = 6). The immunohistochemistry (IHC) staining was performed using anti-β-catenin (1:200, Abcam) and was visualized by the Mouse and Rabbit Specific HRP/DAB (ABC) Detection IHC Kit (Abcam), according to the manufacturer’s instructions. The digital photomicrographs were taken from representative areas using a digital camera. Deparaffinized and hydrated sections were performed immunofluorescence (IF) staining. After heat-mediated antigen retrieval for 2 min in citrate buffer, sections were blocked with 1% BSA buffer for 20 min and incubated with anti-β-catenin (1:200, Abcam), anti-COX2 (1:200, Abcam), anti-Ki67 (1:200, Abcam) and anti-CD31 (1:100, Invitrogen, Carlsbad, CA, USA) overnight at 4°C. After PBST washing, sections were incubated with Alexa Fluor™ 647 goat anti-rabbit IgG (1:500, Invitrogen), Alexa Fluor™ 488 goat anti-rabbit IgG (1:500, Invitrogen) for 1 h at room temperature. Following PBST rinsing, the stained slides were mounted with Fluoromount-G^®^ (SouthernBiotech, Birmingham, AL, USA). Stained sections were observed by a confocal microscope Leica TCS SP6.

### Statistical Analysis

The statistical analysis was performed using Prism 8.0 (Graphpad). Statistical significance was determined by ANOVA or t-test. A *p*-value less than 0.05 was considered statistically significant**.**

## Results

### Characterization of Milk Exosome and Exosome-free Milk

**FIGURE 2:**
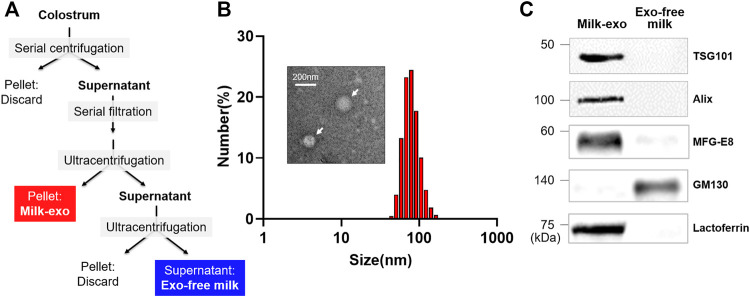
Preparation and characterization of colostrum-derived exosome (Milk-exo). **(A)** Scheme of the preparation procedure of Milk-exo and Exo-free milk. **(B)** Size distribution diagram and representative TEM image of Milk-exo. **(C)** Western blot analysis of common exosome markers (Tsg101 and Alix) and milk specific proteins (MFG-E8 and lactoferrin). GM130 was used as a negative control.

Prior to investigating the hair regeneration ability of colostrum-derived exosomes, milk exosome (Milk-exo) was isolated from colostrum as previously described method with minor modification (Figure 2A) (Vaswani et al., 2017). We also prepared an exosome-free fraction (Exo-free milk) as a control group in which exosomes were completely removed from colostrum through an additional centrifugation step. The size of the extracted Milk-exo and Exo-free milk was analyzed through dynamic light scattering (DLS) analysis (Figure 2B). Milk-exo showed a size distribution of about 50 ∼ 100 nm as previously reported (Sedykh et al., 2020), whereas >10 nm nano-sized vesicles were not observed in Exo-free milk (data not shown). The shape of Milk-exo observed with a transmission electron microscope (TEM) was a round sphere. Furthermore, western blot analysis showed that the exosome markers TSG101 and Alix were identified in Milk-exo samples, and GM130, a Golgi marker commonly used as a negative control for exosome protein markers, was detected only in Exo-free milk (Figure 2C). In addition, MFG-E8, also known as lactadherin, and lactoferrin, which are known to be abundant in milk, were highly loaded in Milk-exo (Ripolles et al., 2018; Superti, 2020). In particular, interestingly, it was found that lactoferrin, which is known to prevent hair loss and promote the growth of dermal papilla (DP) cells through the Erk/Akt and Wnt signaling pathway, is overexpressed only in Milk-exo (Li H. et al., 2019; Huang et al., 2019).

### *In Vitro* Effect of Milk-Exo on the Proliferation of Dermal Papilla Cells

**FIGURE 3:**
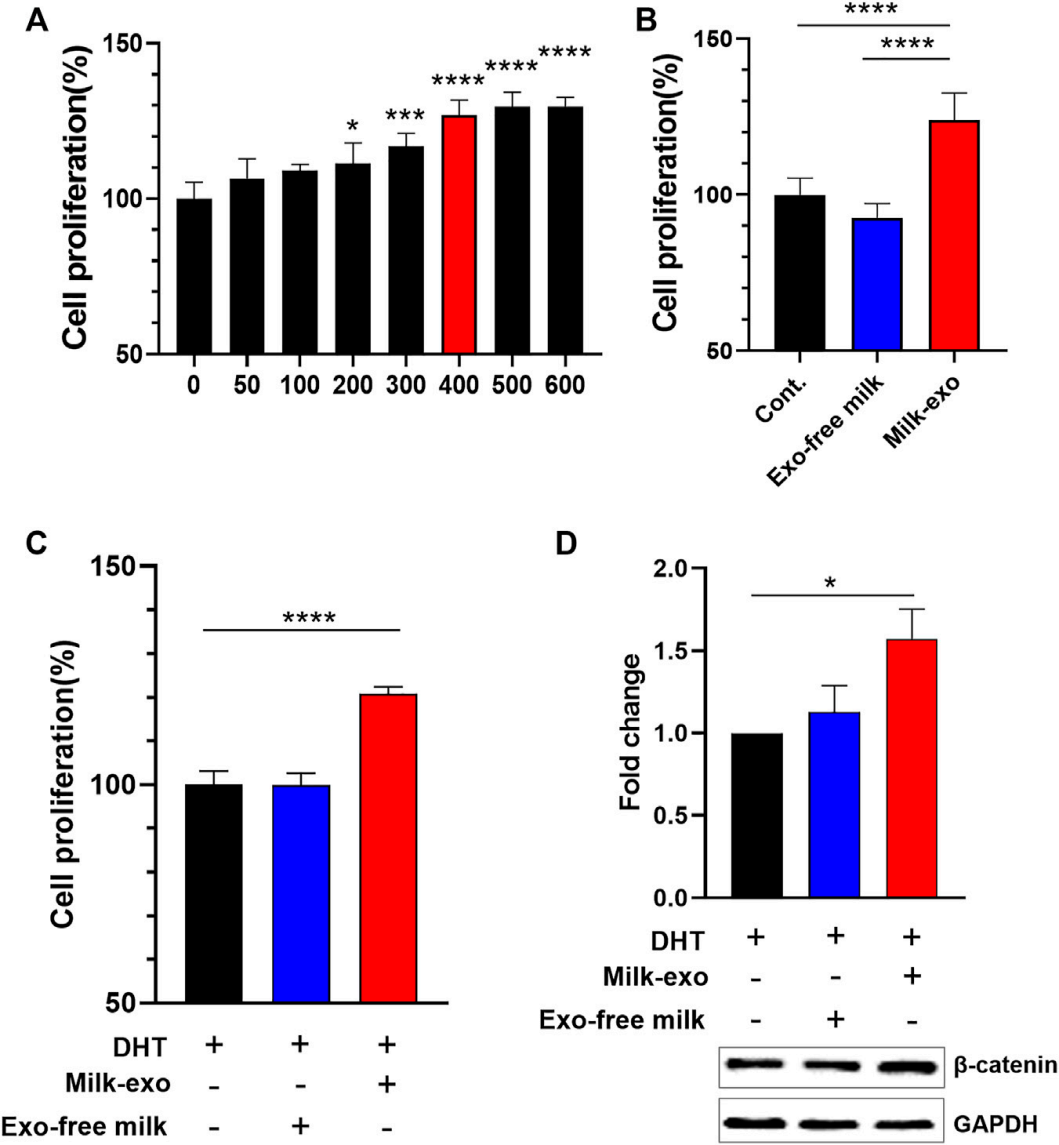
Effect of Milk-exo on *in vitro* proliferation and β-catenin expression. **(A)** Proliferation analysis of DP cells in response to varying concentration of Milk-exo for 24 h (*n* = 6). **(B)** Proliferation analysis of DP cells treated with Milk-exo and Exo-free milk at a concentration of 400 μg/ml for 24 h (*n* = 6). **(C)** Analysis of DP cell proliferation pattern after dihydrotestosterone (DHT) treatment (*n* = 6). **(D)** Western blots on *β*-catenin and GAPDH in DP cells after Milk-exo and Exo-free milk treatment for 24 h (*n* = 3). All data are presented as mean ± SEM (*p < 0.05, ***p < 0.001, and ****p < 0.0001 versus control).

Next, we investigated the influence of Milk-exo on the proliferation of DP cells. First, the cellular uptake efficiency of exosomes in DP cells was examined using confocal microscopy with Milk-exo labeled with Cy-5.5 (Supplementary Figure S1). The confocal image showed that Milk-exo was gradually absorbed by DP cells over time, and almost all exosomes were uptaken within 12 h. Further, we examined the change in the proliferation rate of DP cells according to the concentration of Milk-exo (Figure 3A). As the concentration of Milk-exo increased, the proliferation rate of DP cells gradually increased and saturated at a concentration of about 400 μg/ml. However, there was no change in the cell proliferation rate in the DP cell group treated with the same concentration of Exo-free milk compared to the control group (Figure 3B). In addition, we explored whether Milk-exo could rescue the decrease in DP cell growth caused by the DHT treatment, a male hormone known as a representative cause of hair loss, with its ability to enhance the proliferation rate of DP cells (Figure 3C) (Ustuner, 2013). While Exo-free milk-treated group had no effect, surprisingly, the Milk-exo-treated group showed improved DP cell proliferation compared to the control group. Ultimately, the expression level of *β*-catenin, which promotes the regulation and differentiation of keratinocytes as well as the induction of DP cell to the anagen phase, was compared by Western blot analysis (Figure 3D). Similar to the results of proliferation of DP cells, the expression of *β*-catenin in the group treated with Milk-exo was increased by about 1.5 compared to the control group despite the DHT treatment. Collectively, these results suggest that Milk-exo may affect hair growth by enhancing the proliferation of DP cells and the expression of *β*-catenin.

### Effect of Milk-Exo on Dorsal Hair Re-Growth in C57BL/6 Mice

**FIGURE 4:**
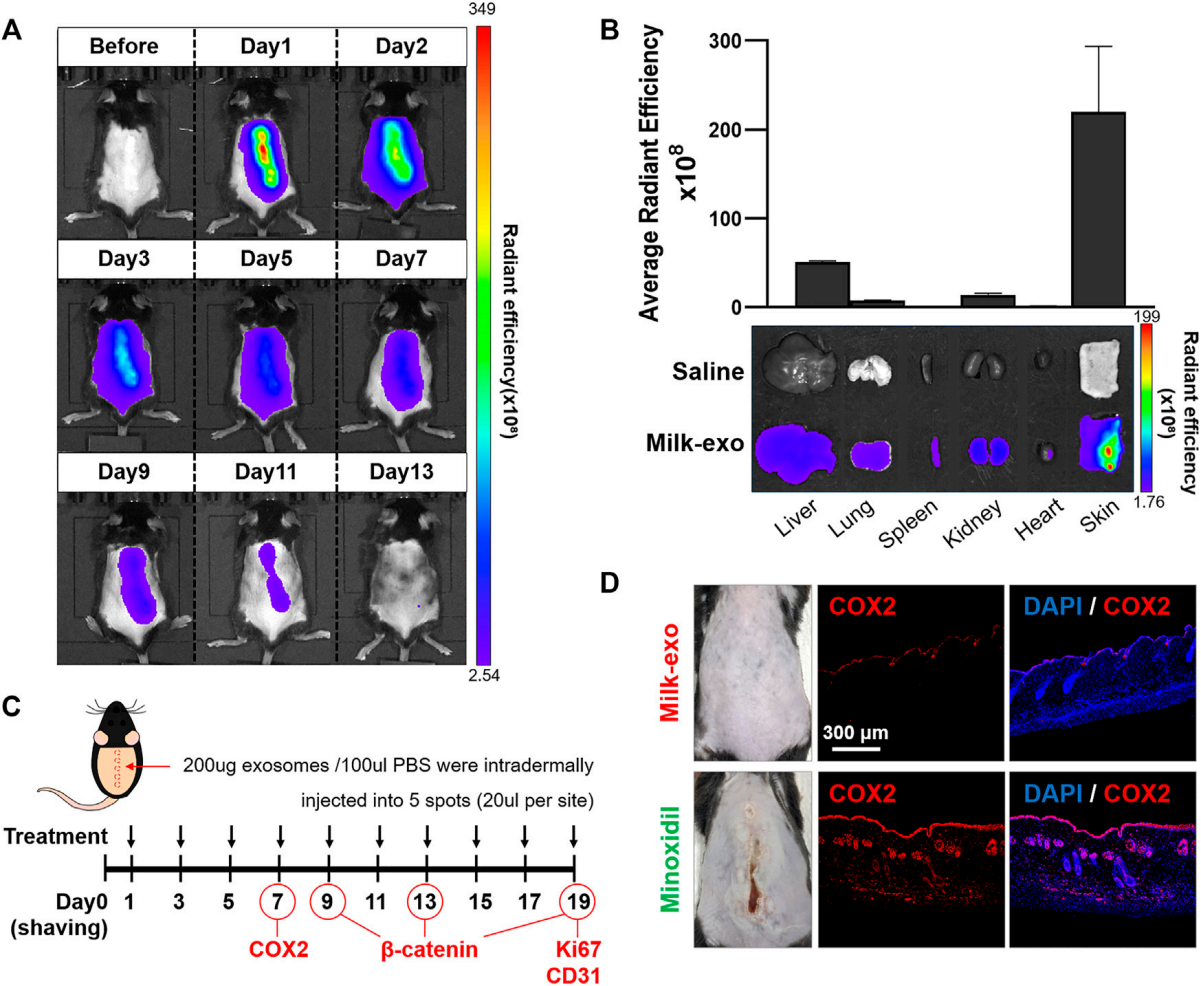
*In vivo* biodistribution of Milk-exo and expression of COX2 in skin tissue. **(A)** Real-time *in vivo* imaging of Cy5.5-NHS labeled exosome in C57BL/6 mice for 13 days after intradermal injection. The mice were analyzed at the indicated times after intradermal injection of 200 µg per 100 µL of exosomes. **(B)** *Ex vivo* imaging and Milk-exo quantification of the skin and major organs on day 2 after intradermal injection of labeled exosomes (*n* = 3). **(C)** Schedule of Milk-exo treatment (black arrows) and the date of histological analysis (COX2, *β*-catenin, Ki67 and CD31). **(D)** Representative immunostaining images of the expression of COX2 in mice skin tissues treated with Milk-exo and Minoxidil. C57BL/6 mice were sacrificed on day 7 after intradermal injection every 2 days. The nuclei (blue) were stained with DAPI, and Alexa Fluor^®^ 647 conjugated secondary antibodies (red) were used for visualization of COX2.

Prior to examining *in vivo* hair growth ability of Milk-exo, the biodistribution of exosomes was confirmed by intradermal injection of Milk-exo on the dorsal side of C57BL/6 mice (Figures 4A,B). Milk-exo injected into the back of the mouse was remained localized for about 2 days, then gradually dissipated, and all was excreted on the 11th day. The level of Milk-exo was the highest in skin tissues compared to other tissues of mice that were sacrificed 2 days after the intradermal injection. Therefore, *in vivo* experiment was scheduled with Milk-exo injection every 2 days (Figure 4C) (Zhang et al., 2013; Son et al., 2018). Minoxidil, a commercially available drug for hair loss, was used as a positive control. Minoxidil, as the first-line pharmacologic recommendation for most patients with androgenetic alopecia, has been proven to be efficient in slowing hair loss by a number of double-blind, randomized, and case-control studies. However, minoxidil treatment is known to induce several adverse effects including contact dermatitis, pruritus, dryness, and facial hypertrichosis even when applied topically. Indeed, the intradermal injection of minoxidil caused severe skin irritation, and immunostaining of skin tissues confirmed that the expression of COX2, which is related to the inflammatory response, was very high (Figure 4D). However, the expression of COX2 was hardly observed in mice skin tissues treated with Milk-exo even after the three consecutive injections.

**FIGURE 5:**
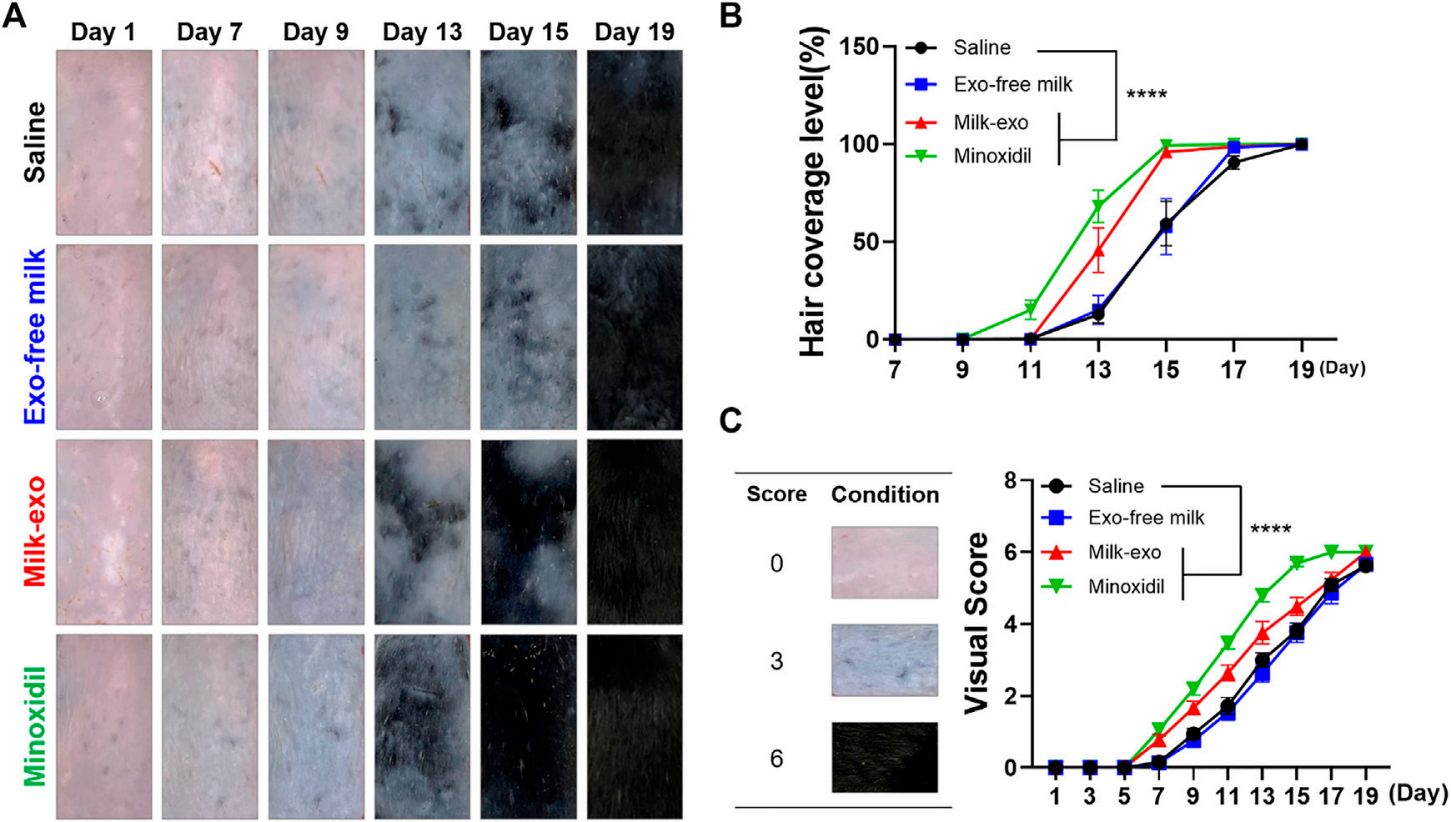
Hair regeneration effect of milk exosomes in C57BL/6 mice. **(A)** Representative images of hair regeneration after intradermal injection of saline, Exo-free milk, Milk-exo (200 μg per 100 μL), and topical application of 2.5% minoxidil. Each reagent was administrated every other day for 3 weeks. **(B)** Quantification of hair coverage level (*n* = 8, ****p < 0.0001 versus saline, two-way ANOVA, Dunnett’s multiple comparisons test). **(C)** Mean visual score measurements (*n* = 8; ****p < 0.0001, two-way ANOVA, Dunnett’s multiple comparisons test).

Next, *in vivo* hair regeneration effect of Milk-exo was monitored over about 3 weeks period after shaving the dorsal hair of mice with animal clippers. The severe skin reactions from minoxidil injection inhibited the intradermal group, and topical administration of minoxidil (at a concentration of 2.5%) were used for the experiments Hair coverage level was used as a direct indicator of hair regeneration, and clinical visual scores were evaluated on a scale of 0–6 (Park et al., 2012). The rate of hair regeneration was not significantly different between groups until around the 7th day, but from the 9th day, the dorsal skin darkened and hair began to appear in the Milk-exo and minoxidil-treated groups (Figure 5A). On day 13, the Exo-free/saline group had an average of only 25% fur coverage, and the fur coverage of the Milk-exo group was approximately 50%. The highest hair coverage was seen on day 15 in the Milk-exo treatment group, at the level comparable to minoxidil treatment (Figure 5B). Visual scoring data also showed consistent results (Figure 5C).

### Acceleration of Telogen-to-Anagen Transition by Milk-Exo

**FIGURE 6:**
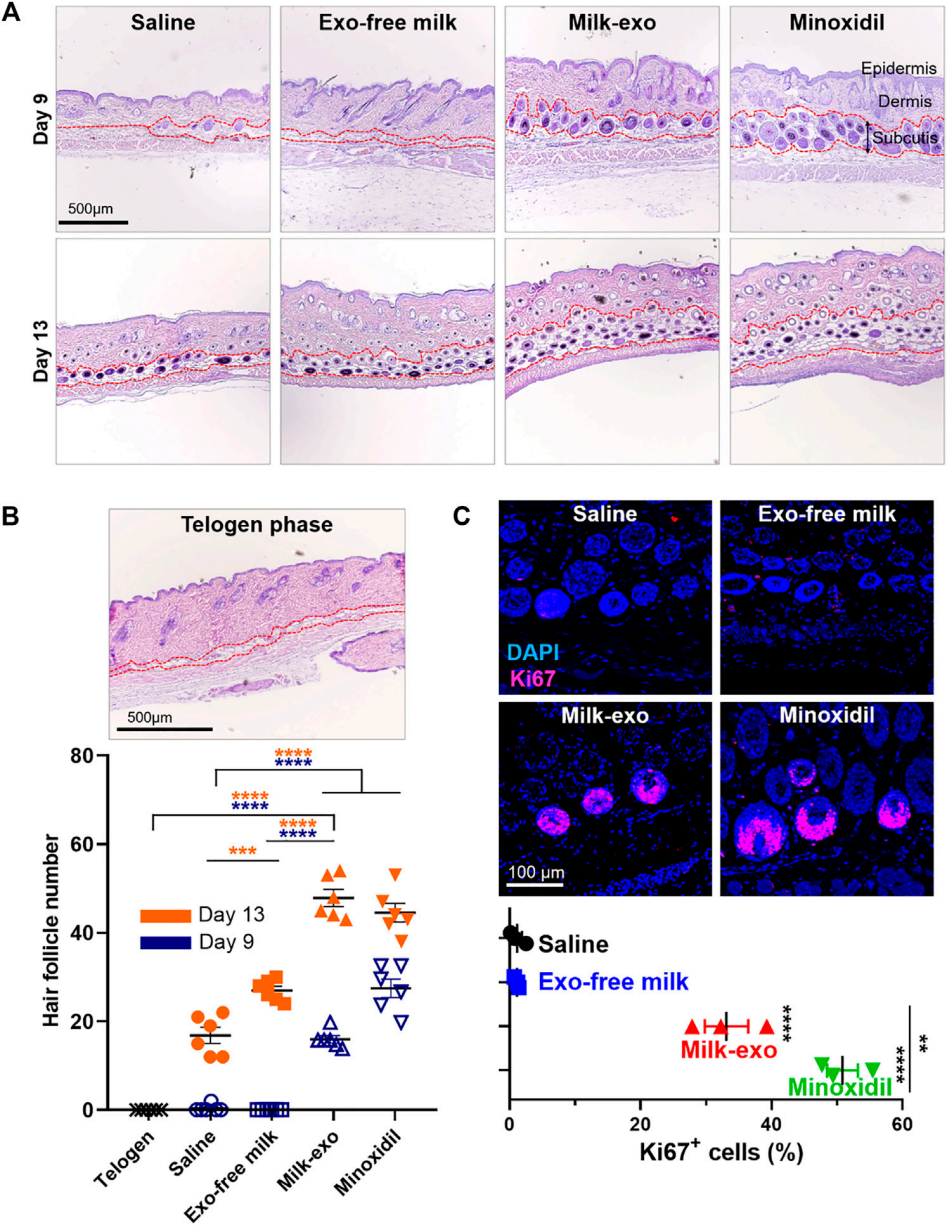
Histological investigation of the acceleration of telogen-to-anagen transition by milk exosomes. **(A)** Representative hematoxylin-eosin (H&E) images of the dorsal skin section for each group on days 9 and 13. **(B)** Representative image of telogen phase at day 1 after shaving and quantification of hair follicle for each groups from three tissue samples (*n* = 6). **(C)** Representative images and quantification graphs showing the expression of Ki67 on day 19 after different treatments from three tissue samples (*n* = 3). All data are presented as mean ± SEM (**p < 0.01, ***p < 0.001 and ****p < 0.0001).

To determine the effect of Milk-exo on hair follicles growth, hematoxylin and eosin (H&E) staining was performed on dorsal skin sections after 9 and 13 days of treatment (Figure 6A). In the stained section of the dorsal skin on day 9, typical anagen features such as thickened subcutis and large hair bulbs were found in the Milk-exo and minoxidil treatment groups. In addition, in the dorsal skin tissue section at day 13 of treatment, the Milk-exo treatment group significantly increased the number of hair follicles compared to the saline/Exo-free milk treatment group (Figure 6B). Furthermore, we performed immunofluorescence staining for Ki67, a proliferation marker of DP cells (Figure 6C). Ki67 signals in the nucleus of the hair matrix cells were found to be 50% and approximately 35% for minoxidil and Milk-exo, respectively. In association with angiogenesis, CD31, which is highly expressed in early anagen, was additionally identified (Mecklenburg et al., 2000; Li et al., 2019b), and the Milk-exo treatment group showed the highest expression rate (Supplementary Figure S2). This result may be due to recently reported angiogenesis-related proteins such as MFG-E8, which were highly expressed in Milk-exo identified in the characterization experiments (Uchiyama et al., 2014; Zhang et al., 2021). Taken together, these data indicated that Milk-exo *in vivo* could further enhance hair matrix proliferation and accelerate the telogen-to-anagen transition of hair follicles (Sugaya and Hirobe, 2014; Bai et al., 2015).

**FIGURE 7:**
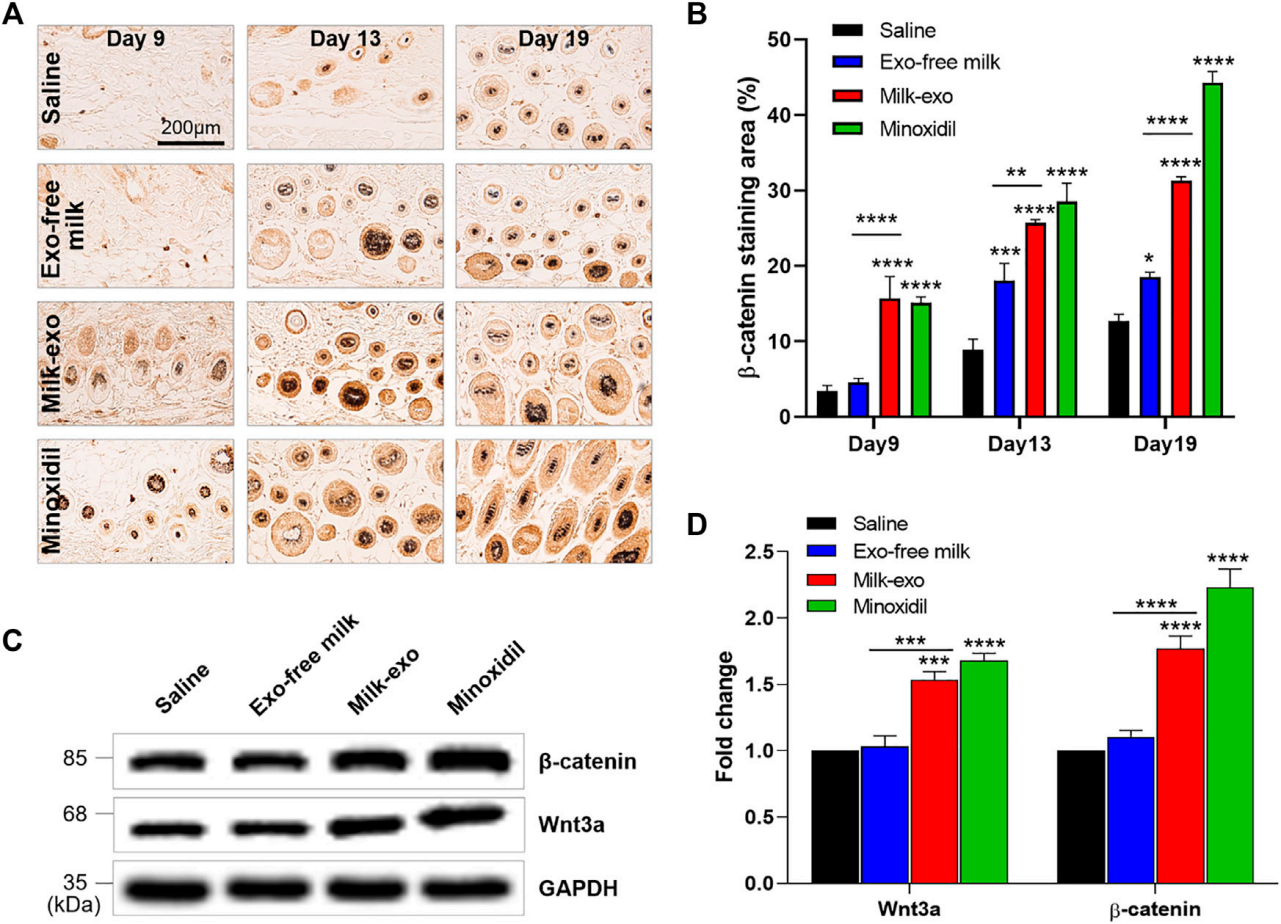
Milk-exo activates Wnt signaling for the transition to the growth phase. **(A)** Immunohistochemical analysis of the expression of *β*-catenin in hair follicles section (day 9, day 13 and day 19). **(B)** Quantification of relative expression of *β*-catenin from five tissue samples (*n* = 4). Data are presented as mean ± SEM (*p < 0.05, **p < 0.01, ***p < 0.001 and ****p < 0.0001). **(C)** Western blot analyzing *β*-catenin, Wnt3a and GAPDH protein content in the dorsal skin on day 9. **(D)** Quantification of western blot protein levels by group. Data are presented as mean ± SEM (n = 3; ***p < 0.001 and ****p < 0.0001).

In particular, *β*-catenin, known as the primary initiator of the anagen phase, is a major regulator of hair follicle growth (Bejaoui et al., 2019). We investigated whether Milk-exo influences the expression of *β*-catenin using immunohistochemical analysis (Figures 7A,B, Supplementary Figure S3). Consistent with the previous data, *β*-catenin was expressed in the Milk-exo and minoxidil-treated groups on the 9th day after treatment. Specifically, quantification data of *β*-catenin–positive (β-catenin+) cells showed that *β*-catenin expression by Milk-exo treatment was gradually increased from day 9 (18%) to day 19 (30%).

Among a variety of inter-/intracellular signaling molecules, Wnt signaling acts a crucial role in hair follicle development *via* triggering the entry into the anagen phase. The secreted Wnt proteins bind with the Frizzled receptor, thereby stabilizing hypophosphorylated *β*-catenin by inhibiting glycogen synthase kinase-3β-mediated protein degradation as well as promote self-renewal of stem cells (Huelsken et al., 2001; Yang et al., 2012). As expected, the expression of Wnt3a was increased in the Milk-exo and minoxidil-treated groups (Figures 7C,D). As with the previous histological data, the expression level of *β*-catenin in each group also showed a similar trend to that of Wnt3a. Collectively, Milk-exo could activate Wnt/β-catenin signaling that enables to induce the transition to the growth phase.

### Characterization and Hair Regeneration Ability of Lyophilized Milk-Exo

**FIGURE 8:**
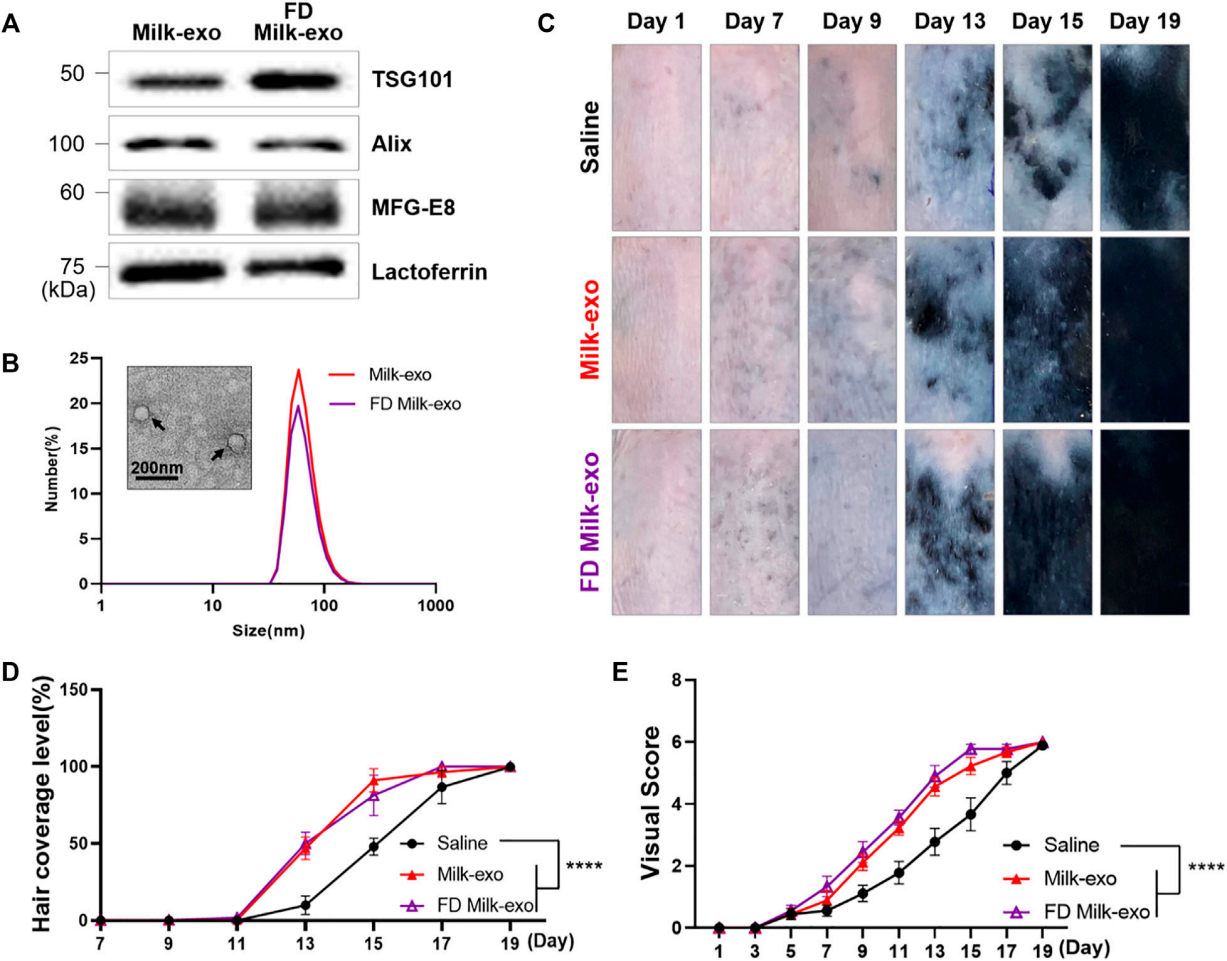
Characterization of lyophilized Milk-exo and conservation of *in vivo* hair growth ability. **(A)** Western blot analysis of exosome markers (TSG101, Alix) and milk specific proteins (MFG-E8 and lactoferrin). **(B)** Representative TEM image and size distribution diagram of lyophilized Milk-exo. **(C)** Representative images of hair regeneration after intradermal injection of saline, Milk-exo and FD Milk-exo. **(D)** Quantification of hair coverage level (*n* = 3, ****p < 0.0001 versus saline, two-way ANOVA, Dunnett’s multiple comparisons test). **(E)** Mean visual score measurements (*n* = 3; ****p < 0.0001, two-way ANOVA, Dunnett’s multiple comparisons test).

Milk, which contains various nutrients, is an ecosystem-optimized diet that humans have been consuming for thousands of years. The bioactive components of milk pass through the digestive system where encounters multiple stress factors such as shifting pH and are well absorbed into the body. Here we investigated whether Milk-exo, which has undergone freeze-drying, maintains its physicochemical properties and hair regeneration activity (Figure 8). First, Milk-exo powder prepared by freeze-drying at -85°C and 58 mTorr for 2 days were again dissolved in PBS (FD Milk-exo), and their size, morphology, and the expression of exosome-specific markers were evaluated. FD Milk-exo also expressed exosome markers (TSG101 and Alix) and milk-specific proteins (MFG-E8 and lactoferrin), and their size and shape did not change even after lyophilization (Figures 8A,B). Furthermore, the hair regeneration ability of FD Milk-exo was evaluated in the C57BL/6 mouse model (Figures 8C–E). FD Milk-exo showed remarkable hair growth ability compared to saline from the 13th day, and this hair regeneration efficiency was similar to that of Milk-exo without freeze-drying.

## Discussion

Advances in our understanding of the hair fiber production regulated by cyclical hair follicle function have opened the door to treatments to safely and effectively enhance hair growth. In the hair follicle cycle, the transition from the telogen to the anagen phase is the trigger to promoting hair regrowth. In this study, Milk-exo were proved to be efficient Wnt/β-catenin activators and identified as important for promoting human hair follicle DP cell proliferation and hair growth. Notably, we also confirmed that lactoferrin is highly expressed in Milk-exo. Lactoferrin, an iron-binding glycoprotein found in body fluids including milk, exerts effects on the stimulation of DP cell proliferation and hair growth. Based on the fact that the expression of lactoferrin is significantly low in chronic telogen effluvium, lactoferrin supplement is expected to be effective in the treatment of non-scarring alopecia (Huang et al., 2019; Milad et al., 2020). Therefore, lactoferrin, known as a hair growth promoter and anti-aging agent, might be one of the key regulators for hair growth in Milk-exo.

However, it is difficult to explain the complexity of the hair growth mechanism with a lactoferrin protein alone. Bovine colostrum contains a rich mixture of bioactive ingredients that can provide significant nutritional and immunological health benefits to humans, and support continues the process of cellular renewal. In particular, the bioactive components such as various miRNAs that post-transcriptionally regulate critical biological processes are abundant in colostrum-derived exosomes (Ross et al., 2021). Recent evidence has shown that exosomes from colostrum are also enriched with extremely high amounts of bioactive proteins related to cell growth and immune response (Samuel et al., 2017). Therefore, the “bioactive cocktail” within Milk-exo would be a more suitable answer for promoting hair regeneration, than a simple single protein.

Recently, exosomes derived from cultured mesenchymal stem cells or human hair follicle DP cells are increasingly highlighted as hair restoration materials (Rajendran et al., 2017; Hu et al., 2020). Although these cell-derived exosomes exhibit effective hair regeneration ability, there are many barriers to overcome for clinical application in that the time and cost required for cell culture is very high and the yield is very low. In addition, since stem cell-derived exosomes can act as a potential driver of genetic reprogramming, specific mechanisms should be fully elucidated for biological safety (Jayaseelan and Arumugam, 2021). On the other hand, our data demonstrated that Milk-exo has a comparable hair regeneration ability as minoxidil and is a safe biomaterial without other adverse effects such as dermatitis. Oral ingestion of bovine colostrum particularly showed no side effects in clinical research even on repeated application of high doses, except lactose intolerance (Struff and Sprotte, 2008). Moreover, milk-derived exosomes have a very high yield compared to animal cell-derived exosomes, which has economic advantages for drug commercialization (Kim et al., 2021).

In addition to improving hair follicle function, milk-derived exosomes might mediate regenerative outcomes in injury and disease states: control inflammation, accelerate and stimulate cell migration and proliferation, mediate wound healing and scar tissue production, increase angiogenesis, and trigger skin rejuvenation (Serra et al., 2017). Additionally, milk-derived exosomes are receiving attention in the pharmaceutical industry because of their additional potential as highly effective drug carriers. Our data showed that there was no change in specific physical properties and activity of Milk-exo that had undergone the freeze-drying process. This could be an important advantage for convenient storage and transportation of exosome-based drugs, supporting facile commercialization. Therefore, in this study, we propose milk exosomes as potential agents to modify and enhance signaling pathways that can induce hair follicle stem cell reactivation, hair cycle, and hair follicle regeneration.

## Conclusion

Our study demonstrated that milk-derived exosomes, particularly colostrum-derived exosomes, induced proliferation of DP cells and accelerated hair regeneration through activation of the Wnt/β-catenin pathway. Compared to commercially available minoxidil, milk-derived exosomes are bio-products with fewer adverse effects due to their high biocompatibility and have advantages as a pharmaceutical product by securing high stability through freeze-drying. Note that if hair follicles are dead, gone, or beyond repair, colostrum-derived exosomes will have the potential to protect and enhance the hair follicles that can be rejuvenated. Therefore, Milk-exo, a safe and biocompatible material could be one of the most efficient modalities to combat hair loss.

## Data Availability

The original contributions presented in the study are included in the article/Supplementary Material, further inquiries can be directed to the corresponding authors.
