# Supplementary material for: Potential of Colostrum-Derived Exosomes for Promoting Hair Regeneration Through the Transition From Telogen to Anagen Phase
Source: Front Cell Dev Biol. 2022 Mar 10;10:815205. doi: 10.3389/fcell.2022.815205 (PMC8960251; doi:10.3389/fcell.2022.815205)
Supplement: Supplementary file 1 [file DataSheet1.DOCX]

Supplementary Material


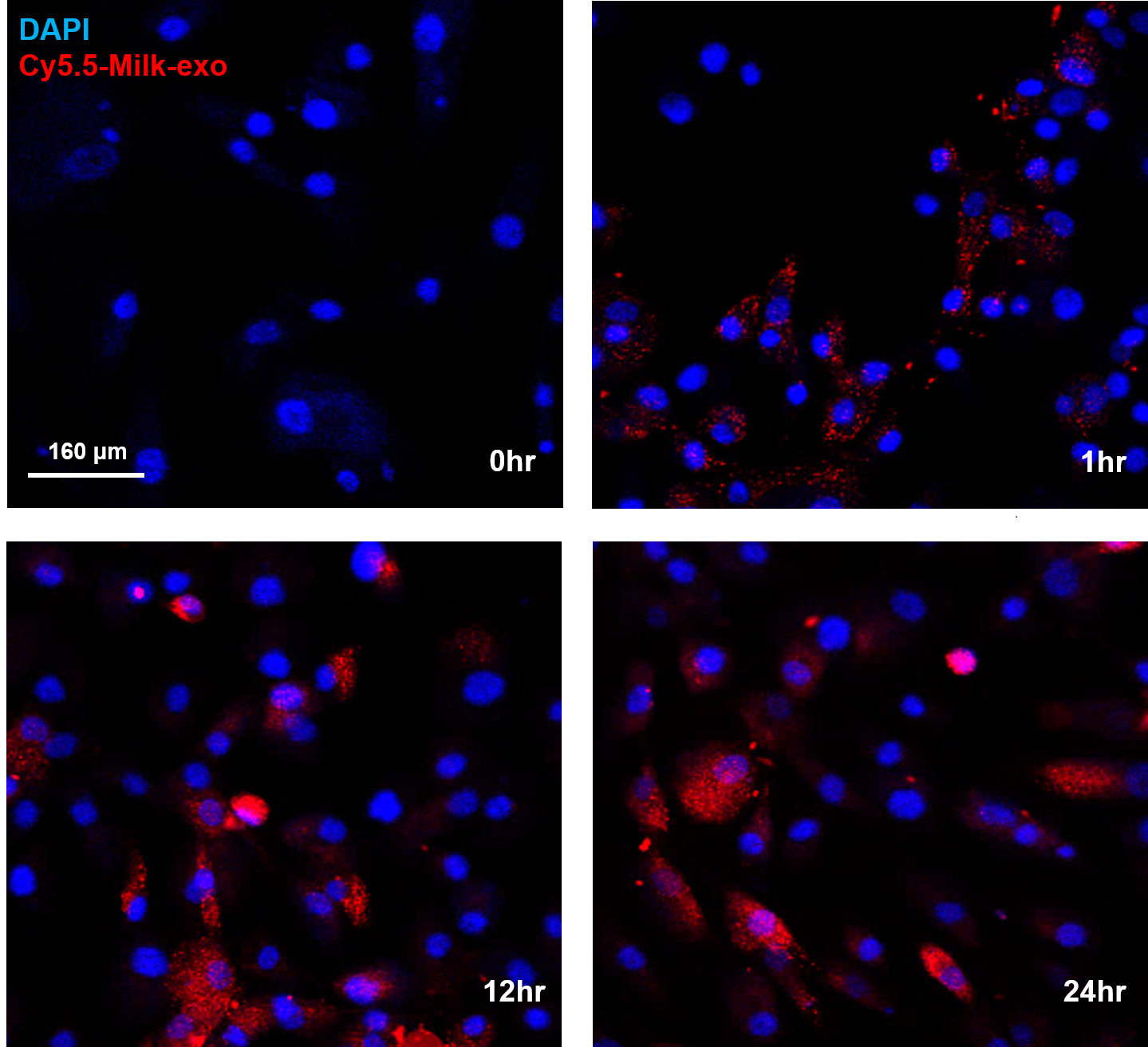


**Supplementary Figure 1.** **Cellular uptake of Milk-exo.** Confocal images of DP cells after 0, 1, 12, or 24 h incubation with 100 μg/ml of Cy5.5 labeled Milk-exo. Images of Cy5.5-labeled Milk-exo (red) with DAPI (blue) were visualized by merging the confocal images.


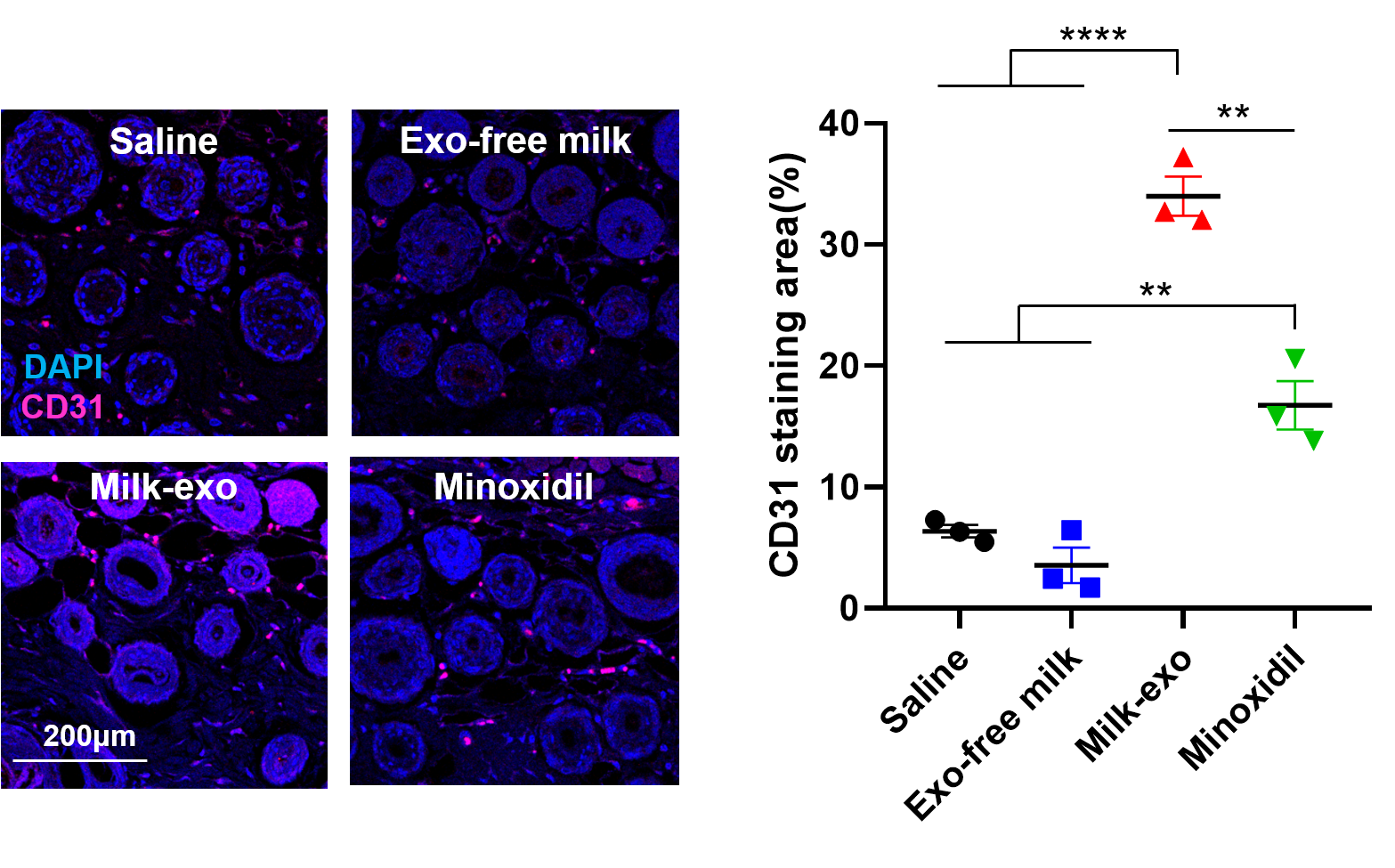


**Supplementary Figure 2. *In vivo* expression of CD31 in skin tissue.** Representative immunostaining images and quantification graphs showing the expression of CD31 on day 19 after different treatments from 3 tissue samples (*n*=3). Data are presented as mean ± SEM (***p* < 0.01 and *****p* < 0.0001)


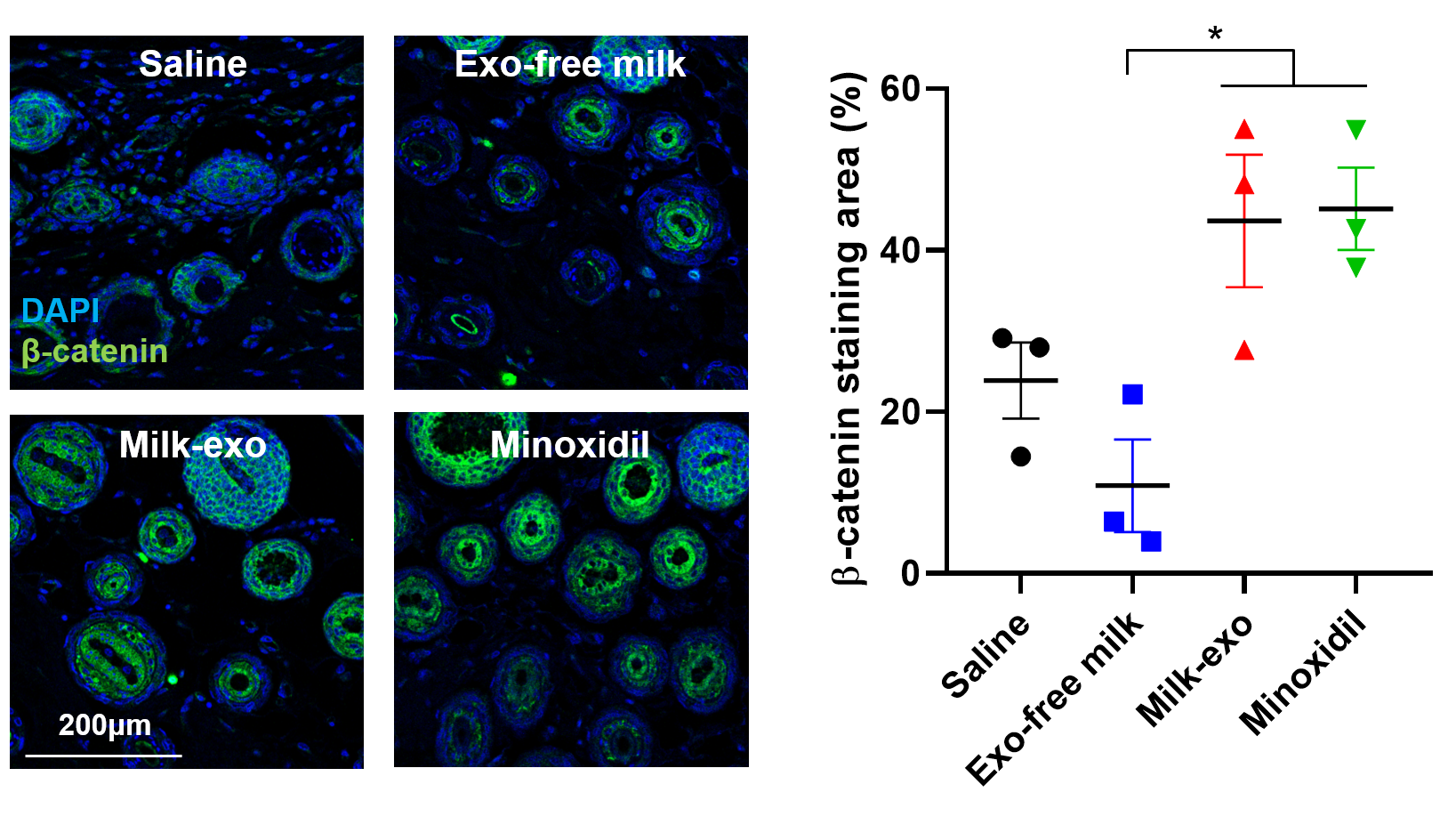


**Supplementary Figure 3.** ***In vivo* expression of β-catenin in skin tissue.** Representative immunostaining images and quantification graphs showing the expression of β-catenin on day 19 after different treatments from 3 tissue samples (*n*=3). Data are presented as mean ± SEM (**p* < 0.05)
